# Learning to generalize

**DOI:** 10.7554/eLife.46651

**Published:** 2019-04-10

**Authors:** André Longtin

**Affiliations:** 1 Department of PhysicsUniversity of OttawaOttawaCanada; 2 Centre for Neural DynamicsUniversity of OttawaOttawaCanada

**Keywords:** electric fish, corollary discharge, generalization, synaptic plasticity, cerebellum, Other

## Abstract

Electric fish are able to take what they have learnt about sensory processing in certain situations and apply it in other situations.

**Related research article** Dempsey C, Abbott LF, Sawtell NB. 2019. Generalization of learned responses in the mormyrid electrosensory lobe. *eLife* **8**:e44032. doi: 10.7554/eLife.44032

To sense our environment our nervous system must be able to distinguish between signals generated by our bodies and signals from our surroundings. Did my head or eyes move, or did my surroundings move? Was my arm moved by myself or by an external agent? To distinguish between these types of signals our nervous system combines a motor control circuit (which tells the body to do something) and one or more sensory circuits (which relay information from various inputs) to create an internal model of the world that allows us to separate the new information in these inputs from information that is not useful.

Understanding how this happens, and solving other amusing puzzles (such as why we can't tickle ourselves), means laying bare the circuitry that the brain uses to learn. In particular, we want to know if these circuits always have to learn to adapt to individual contexts, or can the changes learnt in one context be applied in other contexts that have not been experienced yet? Now, in eLife, Conor Dempsey, Larry Abbott and Nathaniel Sawtell of Columbia University report that weakly electric fish are able to generalize what they learn in one context and apply it in other contexts (Dempsey et al., 2019).

Animals can experience their environment through active or passive sensing. In passive sensing, an animal simply senses stimuli from the environment, including stimuli from other animals. In active sensing, the animal emits signals to sample its environment (e.g., sonar in bats), or modifies its body position to generate responses in primary sensory receptors (e.g., whiskers in rodents). In addition to locating and identifying objects, the signal emitted during active sensing has the potential to interfere with the passive signaling system, which could put the animal's life in danger. However, if the relevant passive sensory circuits are 'warned' about the active sensing pulses in advance, the problem of interference can be countered.

In the 1950s, Erich Von Holst and Roger Sperry independently investigated the 'corollary discharge': this is a copy of a motor command that is sent to a sensory region of the brain every time a motor command is sent to some region of the body. Von Holst and Sperry suggested that a key role of this discharge is to compensate for the effects of unwanted inputs to sensory circuits caused by the motor command. This compensation could be achieved by creating a 'negative image' that cancels out the unwanted inputs, thus leaving behind new signals of interest from the environment.

Weakly electric fish perform both active and passive sensing (Figure 1). They generate a few active pulses per second when they are at rest, and up to 60 pulses per second when they are hunting, with each active pulse inducing an electric field in the water and surrounding objects. Active electroreceptors in the skin detect pulses that have been shaped by nearby objects. In contrast, the passive electrosensing system senses the weak electric signals produced by other animals. However, these signals can become contaminated by the active pulses: indeed, each time an active pulse arrives at a passive electroreceptor, the receptor 'rings' for hundreds of milliseconds during which time any signals from potential prey are masked.

**Figure 1.**
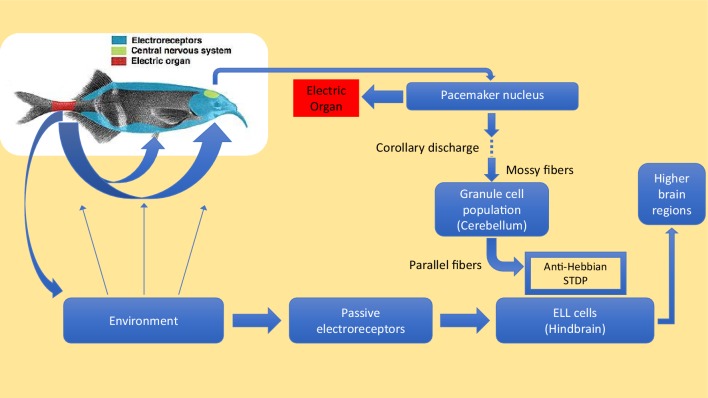
Active and passive sensing in weakly electric fish. Motor commands sent from the pacemaker nucleus in the brain of an electric fish (green region in inset) cause the electric organ in the tail (red region) to emit electric pulses (blue arrows) that travel through the surrounding water. These pulses are shaped by nearby objects and then detected by active electroreceptors (not shown) in the skin. Electric fish also have passive electroreceptors that detect the weak electric signals caused by the muscle movements of other creatures, including the crustaceans that electric fish feed upon: the signals from these receptors are sent to principal cells in a region of the hindbrain called the passive electrosensory lobe (ELL). Electric fish have evolved a sophisticated method to prevent the electric pulses produced by their active sense from interfering with the passive detection of, for example, signals due to body movements made by prey. For each electric pulse it sends, the pacemaker nucleus (top right) also sends a 'corollary discharge' signal along mossy fibers to granule cells in the cerebellum. This signal is processed by the granule cells, and an array of precisely timed signals is sent along parallel fibers to the principal cells in the ELL. These signals alter the strength of the synapses between the parallel fibres and the principal cells in a way that creates a 'negative image' of the original active pulse: the production of the negative image relies on a process known as anti-Hebbian spike time plasticity (STDP). The cells in the ELL use the negative image to subtract the effect of the active pulses from the signals they are receiving from the passive electroreceptors. Any useful information that remains in the signal is forwarded to other regions of the brain. Electric fish tend to emit a few pulses per second when they are at rest, and up to about 60 pulses per second when they are hunting. Dempsey et al. show that electric fish of the species *Gnathonemus petersii* (aka Peter's elephant-nose fish) learn to cancel images at low pulse rates, and are able to generalize this learning to automatically cancel images at the higher pulse rates that they use during hunting.

IMAGE: *G. petersii*: J Jury derivative work: Jmvgpartner [CC BY-SA 3.0].

Pulses emanate from an electric organ in the tail, which receives motor commands from a pacemaker in the brain. Simultaneously, a corollary discharge is sent by that pacemaker to electrosensory cells in the hindbrain (see Figure 1). These cells then subtract the negative image produced by the corollary discharge from the total sensory input received from the electroreceptors (Bell, 1989; Bell et al., 1993; Roberts and Bell, 2000). In an environment that is devoid of features, the signal will be completely nulled by the subtraction. But, if features are present, a signal will remain after subtraction and this signal will be sent to the relevant higher brain regions so that the fish can react as necessary.

To date the cancellation of active sense pulses by electrosensory cells has only been analyzed at low pulse rates, and it was not clear if or how cancellation worked during hunting when pulse rates are higher. Dempsey, Abbott and Sawtell have now recorded the activity of the different cell populations in the corollary discharge pathway for a range of pulse rates. The signals they recorded were then integrated into an expanded version of a computational model they have built (Kennedy et al., 2014). This showed that the generalization of this particular response from low pulse rates to high pulse rates depended on at least two factors: i) the regularization of synaptic plasticity (this well-known machine learning technique avoids reproducing the finer fluctuations in the data, as doing so is detrimental to generalization); ii) nonlinear effects that make the dependence of the activity of the corollary discharge on pulse rate match the dependence of the activity of the electroreceptors on pulse rate.

Similar changes to those observed by Dempsey et al. in the corollary discharge pathway may be involved in how we predict the sensory effects of our own movements and distinguish these from environmental induced effects (Mejias et al., 2013; Straka et al., 2018). The search for synaptic regularization, the effect of natural learning conditions, and the integration of multiple sensory inputs by individual cerebellum cells (Ishikawa et al., 2015) are exciting avenues that will further impact how we think about human motor learning.
